# Deworming utilization among pregnant mothers with at least one antenatal care follow-up in Ethiopia, 2022:- A multilevel analysis

**DOI:** 10.1371/journal.pone.0279967

**Published:** 2023-01-20

**Authors:** Berihun Bantie, Gebrie Kassaw Yirga, Yeshiambaw Eshetie Ayenew, Ahmed Nuru Muhamed, Sheganew Fetene Tassew, Yohannes Tesfahun Kassie, Chalie Marew Tiruneh, Natnael Moges, Binyam Minuye Birhane, Denekew Tenaw Anley, Rahel Mulatie Anteneh, Anteneh Mengist Dessie

**Affiliations:** 1 Department of Comprehensive Nursing, College of Health Science, Debre Tabor University, Debre Tabor, Northwest Ethiopia; 2 Department of Nursing, College of Medicine and Health Science, Wolkite University, Welkite, Southern Ethiopia; 3 Department of Emergency and Critical Care Nursing, College of Health science, Debre Tabor University, Debre Tabor, Northwest Ethiopia; 4 Department of Pediatrics and Child Health, College of Health Science, Debre Tabor University, Derbre Tabor, Northwest Ethiopia; 5 Department of Public Health, College of Health Science, Debre Tabor University, Debre Tabor, Northwest Ethiopia; University of Uyo, NIGERIA

## Abstract

**Background:**

Soil-transmitted helminthes (STHs) are the major public health problems that affect the health of pregnant women and their incoming newborns. In Ethiopia, about 33.35% of pregnant women were affected by these infections. Utilization of deworming medication during pregnancy is the main strategy endorsed by the World Health Organization (WHO) to reduce the burden of STH-induced anemia and its related complications. However, information related to the coverage and its individual as well as community-level factors on the utilization of deworming medication among pregnant mothers with at least one antenatal care (ANC) visit is limited in Ethiopia.

**Methods:**

A nationwide population-based cross-sectional study was conducted from January 18 to June 27, 2016. The information was obtained from the 2016 Ethiopian Demographic Health Survey (EDHS 2016), which can be accessed at: https://www.dhsprogram.com. A weighted sample of 4690 pregnant women selected using a two-stage stratified cluster sampling technique was included in the final analysis. A Multi-variable multilevel binary logistic regression model was fitted to identify the determinants of the utilization of deworming medication during pregnancy. Log-likelihood ration (LLR), deviance and Akaike’s Information Criterion (AIC) were used to select the best fitted model in the multilevel analysis. Statistical significance was declared at p-value <0.05.

**Result:**

From a total of 4690 mothers included in the final analysis, only 365 (7.8%) of them utilized deworming medication in pregnancy. After controlling for confounding effects, having four or more Antenatal care (ANC) visits, having functional working status, intake of iron folic acid (IFA) tablets and coming from a community with a low poverty level increases the odds of utilization of deworming medication during pregnancy.

**Conclusion and recommendation:**

In this study, less than one in ten pregnant mothers takes deworming medication. Mothers with less than four ANC visits, who did not receive IFA tablets, who came from a community with a high poverty level, and mothers with no good functional status were at the greatest risk of not receiving deworming medication during pregnancy. Sustained efforts need to be undertaken to increase the socioeconomic status of the community and to scale up the health care utilization behaviors of pregnant mothers.

## 1. Introduction

Soil transmitted helminthes (STH) infections are common public health problems that impacts the health of reproductive age women’s and children’s [1, 2]. More than 1.5 billion people (24%) of the world’s population were infected by STH infection, with the greatest number occurring in low and middle-incomes [1–3]. Women of reproductive age, preschool-age children, and school-age children have been identified as the three highest risk groups for STH-related morbidities and death [2, 4]. In this aspect, pregnant women are particularly vulnerable to hookworms, and it is estimated that each year, approximately 44 million pregnancies are affected by STH globally [5]. Similarly, an estimated 30% and 70% of pregnant women in Sub-Saharan Africa and in Ethiopia, respectively are also affected by soil-transmitted helminthes infections [6, 7].

Helminthiasis infection during pregnancy poses a serious threat to the health of the mother, the fetus, and their newborns [6, 8–11]. For instance, STHs infections are the leading cause of iron deficiency anemia (IDA) during pregnancy [5, 12]. Over 50% of pregnant women in low and middle income countries (LMIC) suffer from IDA [13–15]. Anemia during pregnancy simultaneously leads to infection, stillbirth/miscarriage, and adverse poor feto-maternal outcomes including preterm birth, low birth weight, and increased neonatal or child mortality [13, 14, 16–18]. In this regard, Ethiopia has been registered as one of the top five countries in the world with the highest neonatal mortality rate (33/1000 live births), which in turn accounts for 58% of Ethiopia’s disability-adjusted life years (DALYs) in 2022 [19, 20]. A lack of iron also hinder the babies’ mental abilities and development as well as their physical growth [21, 22].

The World Health Organization (WHO), therefore, highly recommends administration of a single dose of albendazole (400 mg) or mebendazole (500 mg) coupled with hygiene education to pregnant mothers in order to prevent STH-related complications for the mother as well as the incoming neonate [23–25]. In this aspect, WHO had set a target to attain 75% coverage of deworming for pregnant women in 2030 [26, 27]. Deworming drugs are cheap, safe for pregnancy, and effective in reducing morbidity and mortality related to soil-transmitted helminthes [28–30]. Similarly, a large-scale follow-up study also noted that deworming can result in a 14% and 11% decrease in the risk of neonatal mortality and low birth weight, respectively [31]. In addition, deworming pregnant women and children significantly increases their hemoglobin level and nutritional status, which indirectly results in a huge reduction in maternal and neonatal mortality [32–34]. Large-scale anti-helminthic drug distribution programs, on the other hand, were entirely focused on deworming preschool and school-age children’s. In line with this, currently available research shows that the majority of pregnant women did not receive deworming medication throughout their pregnancy. For instance, only 23% (95% CI 19–28%) of pregnant women in 49 STH endemic countries were dewormed during their routine antenatal care follow-up time [35]. Similar studies conducted in Cameroon also reported that only 29.8% of pregnant married women received deworming medications [36]. Indeed, a number of factors affect the utilization of deworming medication during pregnancy [35–38]. Women’s educational status, age, occupation, economic status, exposure to media, and having optimal ANC visits were a few of them.

Although deworming is the mainstay control strategy for STH-induced anemia and its related complications in pregnancy, data regarding the coverage and individual as well as community-level factors affecting the utilization of deworming medication among pregnant mothers with antenatal care (ANC) in Ethiopia is very limited. Determining the coverage as well as the individual and community level predictors of utilization of deworming medication in pregnant mothers with at least one ANC visit in Ethiopia will have a paramount role in improving national deworming status and averting the complications of STH in pregnancy.

## 2. Methods

### 2.1. Study design, area and period

**Fig 1:**
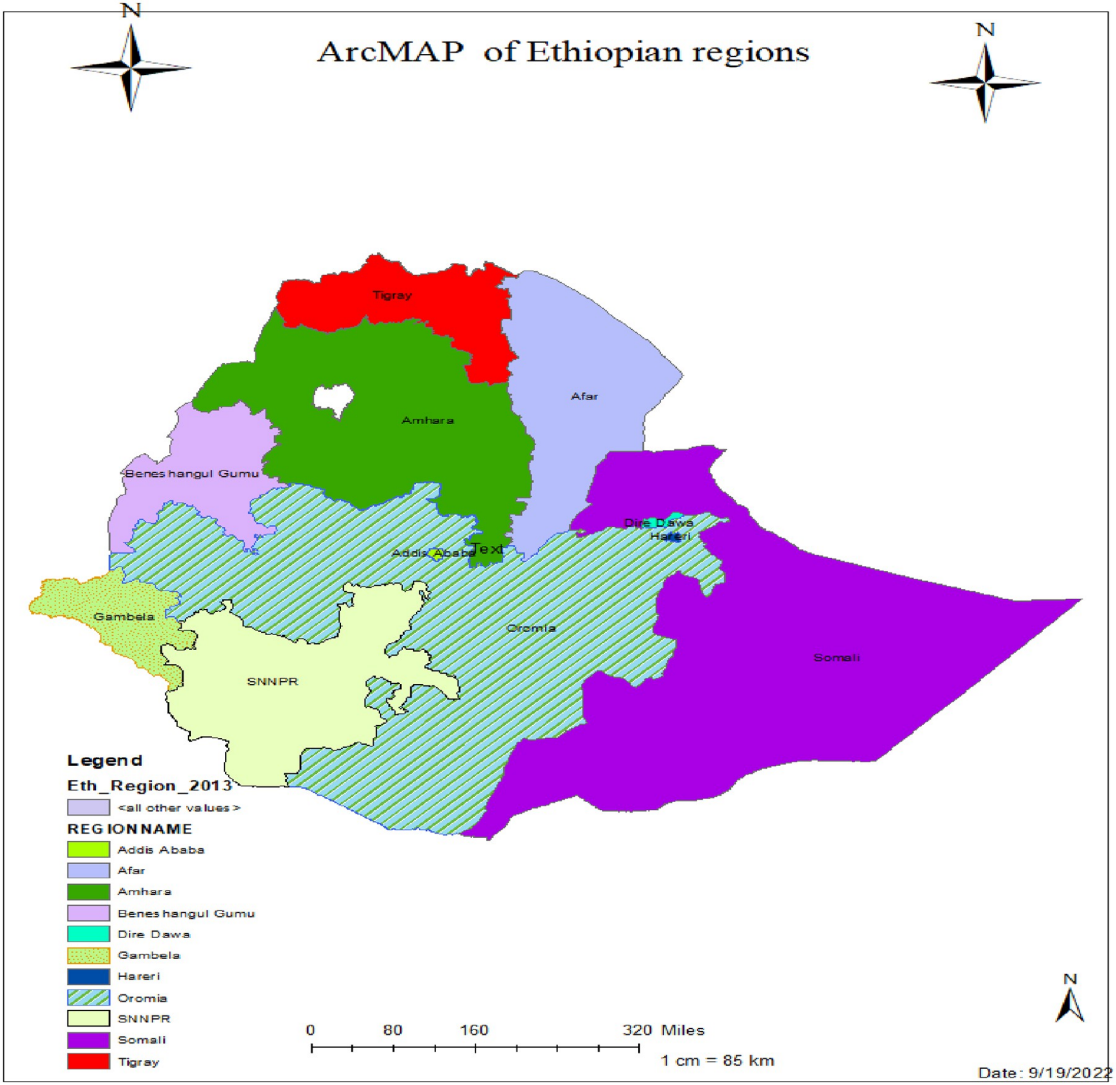
ArcMap of the study areas used to assess utilization status of deworming medication among pregnant mothers in Ethiopia, 2022.

A nationally representative population-based cross-sectional study was conducted from January 18 to June 27, 2016, in Ethiopia using the fourth round of the Demographic Health Survey (EDHS 2016). Ethiopia is the second most populous country in Africa, with a total population of over 117 million [39]. It has a total area of 1,100,00 km2 and lies between latitudes 3° and 15° N and longitudes 33° and 48° E. Ethiopia has nine administrative regions and two administrative cities (Fig 1). The country is also subdivided into 68 zones, 817 districts, and 16,253 kebeles (the lowest administrative unit in the country). Keble’s are further subdivided into census Enumeration Areas (EAs) or clusters, which serve as a sampling frame for surveys. The 2016 survey includes all nine regions and two town administrations in Ethiopia.

### 2.2. Data sources and populations

This study used data from the 2016 Ethiopia Demographic Health Survey (EDHS), which can be obtained from the DHS database www.meauredhs.com. All pregnant women in the last 05 years preceding the survey were the source population, whereas all pregnant mothers with ANC follow up in the last 05 years preceding the survey in the selected EAs were the study populations.

### 2.3. Sample size determination and sampling technique

In the EDHS 2016 survey, a total of 15,683 households (HHs) had eligible women in reproductive groups, and 10,641 under-five children were found in these HHs. In the case of two or more under-five children per household, the most recent births were only considered. Mothers with no ANC follow-up and unknown records of utilization of deworming medication were excluded in the study. Finally, a total of 4658(weighted sample of 4690) women were included in the final analysis.

Regarding the sampling technique, a two-stage stratified cluster sampling design was used to select the final eligible participants. In the first stage, 645 (202 from urban and 443 from rural areas) EAs were randomly selected using a probability proportional to size (PPS) method from the lists of 84915 EAs generated from the 2007 population and housing census. In the second stage, a fixed number of 28 households per cluster were chosen with an equal probability systematic random sampling technique. The detailed procedure regarding the sampling procedure can be obtained from the EDHS, 2016 PDF report [40].

### 2.4. Variables of the study

#### 2.4.1. Dependent/outcome variable

The outcome variable in this study was deworming utilization status of pregnant women’s with at least one ANC follow up. It was categorized into binary (yes/ no) categories.

#### 2.4.2. Explanatory/independent variables

The factors of deworming utilization status were broadly categorized into individual and community-level factors. The individual level variables includes age of the mother, educational status of women and husband, marital status, education of, working, household wealth index, distance to health facility, media exposure, sex of the head of the HH, decision maker status of the women, number of ANC, birth order, IFA intake status and total number of children’s in the HHs.

On the other hand, factors including region, residence, distance to health facility, community poverty level, community women’s education status, and community media exposure status were considered as community level variables.

The community level factors such as community media exposure status, community educational status, community poverty were obtained by aggregating individual-level characteristics at the community (cluster) level. Categorization of the aggregate variables was done as high or low based on the nature of the distribution of the data (using either median or mean).

### 2.5. Operational definitions

Deworming of pregnant mothers was categorized as yes when the pregnant mother takes at least a single dose of albendazole (400 mg) or mebendazole (500 mg) [24]. In the current study, ANC utilization is considered "yes" when women visit a health facility at least once during their last pregnancy, otherwise not. Community poverty status is defined as the proportion of households in the poorer or poorest quintile of each cluster [40]. Community women’s educational level is also defined as the proportion of women who have completed at least a primary level of education in each cluster [40]. Community women’s educational level is also defined as the proportion of women who have completed at least a primary level of education in each cluster [40]. In addition, distance to health facility was defined as the proportion of households with big problem of distance to health facility with each cluster [40]

A decision maker is defined as a person who usually decides on a respondent’s health care utilization. These may include the respondents themselves, their partner/husband, mother-in-law, father-in-law, and someone else.

### 2.6. Data processing and analysis

After the extraction of relevant data, further cleaning, coding, and analysis were done using Microsoft Excel 2013 and STATA V.16 software. A weighted sample using the recommended weighting factor was used to ensure the representativeness of the survey by reducing sampling variability. Summary measures of the variables were obtained using frequency as well as mean or median, depending on the nature of the data. Since the EDHS data had a hierarchical nature (pregnant women’s were nested within a cluster), a model which handles the dependence of observations within a cluster was fitted to identify predictors of deworming among pregnant mothers. Therefore, a multilevel binary logistic regression analysis was computed after observing the value of intra-class correlation (ICC > 5%). First, bi-variable multilevel binary logistic regression models were fitted, and all variables with a p-value less than or equal to 0.25 were fitted to the final model. After recruiting variables for multi-variable analysis, four models were fitted, sequentially: the null model (a model without explanatory variables is used to test random variability in the intercept and to estimate the intra-class correlation coefficient); model 1(containing only individual-level factors); model 2 (containing only community-level factors); and model 3 (which comprises both individual and community-level factors). The log likelihood ratio (LLR), device, and Akaike Information Criterion (AIC) were used to compare and select the best model. The model that has the highest log likelihood or lowest deviance and AIC was taken as the best model.

In addition, the total measure of variation (random effect) of utilization of deworming medication was assessed using ICC, proportional change in variance (PCV), and the median odds ratio (MOR). The MOR was used to determine the heterogeneity between clusters (the second-level variation) by comparing two people from two randomly selected clusters. Proportional change in variance (PCV) is used to assess the total variation attributed to individual and community level factors in the multilevel model as compared to the null model.

Finally, as adjusted odds ratios (AOR) with 95% confidence intervals (CI) was reported in the multi-variable multilevel-logistic regression model and statistical significance was declared at p-value< 0.05. The presence of multi-collinearity among independent variables was checked using a variance inflation factor (VIF>10) through running a pseudo-linear regression analysis.

### 2.7. Ethical considerations

Informed consent from the participants was waived because the study used secondary (2016 EDHS data). Permission to get access to download DHS data was obtained from the major DHS data archivist through a reasonable online request using http://www.dhsprogram.com. In the DHS data, there are no names of individuals or household addresses. The information retrieved was only used for statistical reporting and analysis of our registered research. The EDHS report included detailed information on the procedure for ensuring the ethical issue.

## 3. Result

### 3.1. Individual and community level socio-demographic variables

Related to the socio-demographic profiles of the participants, the majority of them were married (4360, or 93.0%), aged between 25 and 34 (2439, or 52%), lived in rural areas (3823, or 81.5%), and did not attend formal education (2519, or 53.7%). The median age of the mothers was 28 years, with an inter-quartile range (IQR) of 33–24 years. Regarding community level variables, 2,833 (60.4%) of the respondents were from the community with low educational status. In addition, nearly half (2,115, or 45.1%) of the respondents came from the community with a high community poverty level **(**Table 1).

**Table 1:** Socio-demographic and economic characteristics of respondents in 2016 EDHS, Ethiopia (weighted N = 4690).

Variables	Category	Deworming utilization status		Total weighted frequency	%
(N = 4690)		Yes (N = 365)	No (4325)		
Head of the HHs	Male	311	3695	4006	84.4
	Female	54	630	684	14.6
Age category	Age 15–24	82	1143	1,225	26.1
	Age 25–34	219	2,229	2,439	52.0
	Age 35 and above	74	952	1,026	21.9
	Median age = 28 years (IQR = 33–24)				
Religion	Orthodox	179	1,819	1,998	42.6
	Moslem	103	1,423	1,526	32.5
	Protestant	73	974	1,047	22.3
	catholic/traditional/other	10	109	119	2.6
Marital status	Married/ in union	333	4,027	4,360.	93.0
	Not-married/not in union	32	298	330	7.0
Educational status of the respondent	No formal education	180	2,339	2519	53.7
	Primary education	136	1,423	1559	33.3
	Secondary	34	351	385	8.2
	Tertiary and above	15	212	227	4.8
Educational status of the husband	No formal education	125	1,622	1,747	37.2
	Primary education	150	1,649	1,799	38.3
	Secondary	41	467	508	10.8
	Tertiary and above	25	310	335	7.2
	I don’t know	3	20	23	0.5
Working status of the respondent	Working	214	2,982	3,196	68.1
	Not working	151	1,343	1,494	31.9
Region	Tigray	46	433	479	10.2
	Afar	4	33	37	0.8
	Amhara	96	973	1,069	22.8
	Oromia	122	1,450	1,572	33.5
	Somali	3	112	115	2.5
	Benishangul	6	50	56	1.2
	SNNPR	72	1,043	1,115	23.8
	Gambela	1	14	15.	0.3
	Harari	1	12	1 3	0.3
	Addis Abeba	11	181	191	4.1
	Dire Dewa	3	25	28	0.6
Residence	Urban	74	793	867	18.5
	Rural	291	3,532	3,823	81.5
Poverty status of the HHs	Poor	111	1,582	1,693	36.1
	Middle	84	884	968	20.6
	Rich	170	1,859	2,029	43.3
Media exposure status	Not exposed	160	2,527	2,687	57.3
	Exposed	205	1,798	2,003	42.7
Community poverty level	Poor	110	2,005	2,115	45.1
	Rich	255	2,320	2,575	54.9
Community education Status	Low	205	2,628	2,833	60.4
	High	160	1,697	1,857	39.6
Community distance to Health	Big problem	195	2,520	2,715	57.9
	Not big problem	170	1,805	1,975	42.1
Community media Exposure	Low	169	2,491	2660	52.5
	High	195	1,833	2,229	47.5

IQR = Inter-quartile range, HH- Households.

### 3.2. Maternal characteristics

Regarding the ANC utilization status of the mothers, only half (2354 or 50.2%) of them had four or more ANC visits throughout their pregnancy. Additionally, more than one third (1890, or 40.3%) of mothers didn’t receive IFA tablets during pregnancy. One fourth (669, or 14.3%) of the mothers were able to decide solely on their own health care needs, while the rest depended on their husbands or someone else **(**Table 2).

**Table 2:** Maternal characteristics result of respondents in 2016 EDHS, Ethiopia.

Variables	Category	Deworming utilization status		Total weighted frequency	%
(N = 4690)					
		Yes (N = 365)	No (4325)		
ANC visit	ANC1	16	371	387	7.6
	ANC 2 and 3	130	1847	1,977	42.2
	≥ ANC4	219	2,106	2,325	50.2
IFA intake status	Yes	297	2503	2,800	59.7
	No	68	1822	1,890	40.3
Birth order	1^st^	82	1023	1,105	23.8
	2^nd^ -3^rd^	114	1397	1,511	32.2
	4+	159	1915	2,074	44.0
Total living children	0–3	232	2575	2807	59.9
	≥ 04	133	1,750.	1883	40.1
Decision maker	Respondent	67	602	669	14.3
	Other	298	3723	4,021	85.7

Others- Partner/ husband, mother in law, father in law, someone else: IFA- Iron Folic Acid intake.

### 3.3. Deworming utilization status

**Fig 2:**
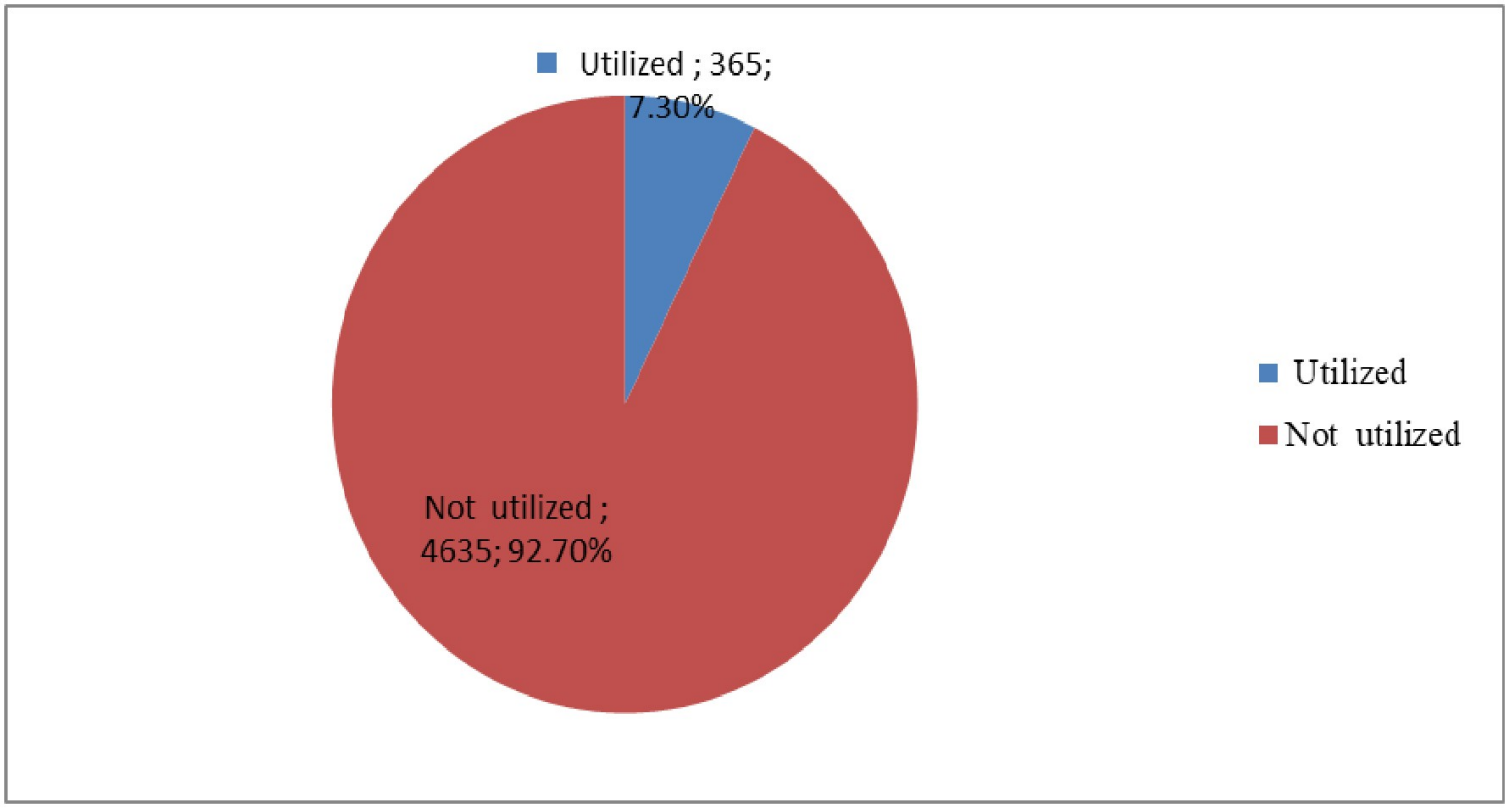
Utilization status of deworming medication among pregnant mothers with at least one ANC visit in Ethiopia, 2022.

In the current study, less than one in ten (7.8%: 95% CI 5.1–10.5%) pregnant women’s utilize deworming medication **(Fig 2)**.

### 3.4. Multilevel binary logistic regression model analysis

As shown in the table below (Table 3), the results of intra class correlation (ICC) in the empty mode showed 23% of the total variation in utilization of deworming medication during pregnancy was attributed to the difference in the community or cluster. Therefore, multilevel (two-level) binary logistic regression model which handles the dependency or correlation of observation with in a cluster was fitted In the multilevel binary logistic regression model, the full model (model III) containing both the individual and community level variables was considered as the best fit model because it has the lowest deviance and AIC value (deviance = 2308, AIC = 2338.237, p-value = 0.0001). The presence of heterogeneity between clusters was also explained by the Median Odds Ratio (MOR). In the full model, the MOR was 2.5, which implies that the odds of utilization of deworming medications was 2.5 among mothers who came from a community with high to low proportion of deworming utilization status. The proportional change in variance (PCV) in the final model also indicates that 2.4% of the variation in the utilization of deworming medication among pregnant mothers observed was explained by both community and individual-level variables.

**Table 3:** Multi-variable multilevel binary logistic regression analysis result of both community and individual level factors associated with utilization of deworming medication in pregnant mothers in Ethiopia, EDHS 2016.

Community and individual level factors	Models			
	Null model	Model I	Model II	Model III
	(AOR 95%CI)	(AOR 95%CI)	(AOR 95%CI)	(AOR 95%CI)
Marital status				
Married/ in union		1		1
Not married		1.24 (0.69–2.24)		1.28 (0.71–2.30)
Religion				
Orthodox		1		1
Muslim		1.23 (0.77–1.98)		1.20 (0.76–1.89)
Protestant		1.12 (0.69–1.84)		1.10 (0.67–1.78)
catholic/traditional/other		1.83(0.52–6.51)		1.77 (0.51–6.13)
Working status of the respondent				
Not working		1		1
Working		1.56 (1.09–2.24)		1.57 (1.09–2.26)*
Media exposure				
Yes		1.49 (1.01–2.03)		1.6 (0.97–2.19)
No		1		1
ANC visit				
ANC 1		1		1
ANC 2 to 3		1.99 (0.76–5.21)		1.983 (0.75–6.51)
≥ ANC 4		2.85 (1.05–7.74)		**2.83 (1.03–7.79**) *
IFA status				
No		1		1
Yes		3.35 (2.11–5.33)		3.42 (2.15–5.43)*
Decision maker				
Respondent		1		1
Others		0.67 (0.41–1.11)		0.67 (0.41–1.83)
Community education level				
Low			1	1
High			0.91(0.65–1.28)	0.76 (0.51–1.13)
Distance to Health facility				
Big problem			1	1
Not Big problem			.89(0.64–1.24)	0.72 (0.50–1.52)
Community poverty level				
High			1	1
Low			1.95 (1.35–2.84)	**1.84 (1.23–2.75**)*
**Measure of random effect (variation)**				
Variance	.9913999	0.97654	0.8624397	0.9688124
ICC (%)	23.2%	23.1%	20.8%	22.8%
PVC (%)	Reference	1.5%	13%	2.3%
MOR	2.57	2.55	2.4	2.5
**Model fitness**				
Log likelihood	-1226.4761	-1159.74	-1218.96	-1154.1187
Deviance	2452.95	2319.48	2437.92	2308
AIC	2456.952	2343.489	2449.92	2338.237

1- Reference category, AOR-adjusted odds ratio, CI- confidence interval, * and old letter—statistically significant variables in the full model.

#### 3.4.1. Determinants of deworming utilization status

Variables with a P-value of <0.25 in the bi-variable multilevel binary logistic regression model were fitted into the final multivariable multilevel binary logistic regression model. In this regard, variables like religion, marital status, working status, ANC visit level, IFA intake status, decision maker on women’s health care needs, household media exposure status, community poverty level, community education level, and distance to the health facility level were computed in the full model (model III). Finally, working status, ANC visit level, IFA intake status, and community poverty level were identified as significant predictors of utilization of deworming among pregnant mothers.

In comparison to their counterparts, pregnant women who took IFA tablets were 3.42 (AOR 3.42, 95% CI: 2.15–5.4) times more likely to use deworming medication In addition, the odds of utilization of deworming medication among pregnant mothers with functional working status was 1.57 times higher (AOR 1.57, 95%CI: 1.09–2.26) when compared with mothers not working status. The utilization of deworming medication in pregnant mothers was 2.83 times (AOR 2.83, 95%CI: 1.03–7.79) more likely in mothers with four or more ANC visits compared with those mothers with only a single ANC visit. The odds of deworming in pregnant mothers who came from a community with a low poverty level was 1.84 (95%CI: 1.23–2.75) times higher than their counterparts.

## 4. Discussion

Deworming pregnant women is the main strategy endorsed by world health organization to reduce the burden and associated complications of STHs. This study was undertaken to investigate the coverage and its community as well as individual level factors of utilization of deworming medication among pregnant mothers with ANC follow-up in Ethiopia. In this study, the coverage of utilization of deworming medication among pregnant mothers was found to be 7.8%. This figure is significantly lower when compared with prior studies conducted in 49 STH endemic countries in the world, in Ghana, Cameron and Tanzania [35–38]. The discrepancy in the proportion of utilization of deworming medication in pregnancy could be due to variation in the time when the DHS data is collected [37, 40, 41], the difference in socioeconomic status, and/or variation in terms of maternal health care utilization status across these countries [42–46]. The very low figure in Ethiopia will further challenge the country’s efforts to reduce neonatal and mortality rates to the WHO threshold level.

In this study, the intake of IFA tablet during pregnancy was found to be significantly associated with the utilization of deworming medication. Those pregnant mothers who received IFA tablets were 3.42 (AOR 3.42, 95% CI: 2.15–5.4) times more likely to utilize deworming medications compared to their counter parts. The possible elucidation for the observed association might be due to the fact that those mothers took IFA were more likely be educated, receive intensive counseling and had optimal ANC visits which all affects the utilization of deworming medication [30, 47].

Deworming medication use during pregnancy is also 2.83 times more likely in mothers who had four or more ANC visits compared to those who had only one ANC visit. This suggests that mothers who had more ANC visits will be more likely to receive adequate information about the utilization of deworming medication during pregnancy, and they may also have enough time to access the medication during their second and third trimesters if they had not taken it during their first ANC visit. This finding is in line with a prior study conducted in Tanzania, Cameroon and in 49 STH endemic countries [35, 36, 38]. Therefore, the quality and duration of ANC services were found to have a significant role in increasing coverage of deworming medication during pregnancy.

In the current study, we also identified that women who came from a community with a low poverty level were more likely to receive deworming medications when compared with their counterparts. The observed association is in line with prior research conducted in Ghana, Tanzania and in 49 STH endemic countries [36–38]. This association could be explained by the fact that women with higher socioeconomic status have better access to health institutions, which increases their health care demands and utilization [45, 48]. Besides, women from the wealthiest communities are more likely to have optimal ANC visits, which further enhance utilization of deworming medications [49–51]. On the other hand, soil transmitted helminthic infections are more prevalent in this economically disadvantaged communities, which necessities a strong and well-organized interventions [52].

Finally, this study also identified that pregnant women with working functional status are almost twice more likely to take deworming medication than their counterparts. This could be explained by the fact that women who do have functional work experience are more likely to perform their daily living activities and to visit health institutions whenever they are in need of health services [53]. In addition, women with working status can easily earn money so that they can afford any payments for their healthcare needs.

### 4.1. Strengths and limitations of study

The major strength of this study is the use of nationally representative data. In addition, the methodology used in the DHS survey increases the generalizability and representativeness of the data. Using multilevel logistic regression analysis, which can easily identify both individual and community-level variables, also plays a pivotal role in designing interventions that target such factors. On the other hand, we would like to remind you that our findings should be considered in light of the following limitations: First, the effect of important variables like the availability of deworming drugs, the level of counseling, and the perception and attitude of mothers towards deworming medications were not investigated. Secondly, due to the retrospective nature of the response, recall bias might be introduced.

### 4.2. Implication of the result of this study

The very low uptake of deworming medication among pregnant mother in this study implies that a substantial number of mothers were affected by STH-related complications, which further increase neonatal and maternal mortality. Therefore, maternal and newborn health programs should give great emphasis towards improving the utilization of deworming medication during pregnancy.

## 5. Conclusions

The finding of this study shows that less than one in ten pregnant mothers with at least one ANC visit utilize deworming medication, which is too far from WHO 2030 target. Mothers with less than four ANC visits, not taking IFA tablets, came from a community with high poverty level and mothers with not good functional status were at most risk for not receiving deworming medication during pregnancy. Sustained effort need to be undertaken to increase the socioeconomic status of the community and to scale up health care utilization behaviors of pregnant mothers.

## Abbreviations

AIC: Akakies information criteria
ANC: Antenatal care
AOR: Adjusted odds ratio
CI: Confidence interval
DHS: Demographic Health Survey
EDHS: Ethiopia Demographic Health Survey
HHs: Households
ICC: Intra cluster correlation
IFA: Iron Folic Acid tablet
IQR: inter quartile range
MOR: Median Odds Ratio
PCV: Proportion of change
STH: Soil-transmitted helminthes
WH0: World Health Organization
